# Relationship Between Isokinetic Lower-Limb Joint Strength, Isometric Time Force Characteristics, and Leg-Spring Stiffness in Recreational Runners

**DOI:** 10.3389/fphys.2021.797682

**Published:** 2022-01-21

**Authors:** Shiqin Chen, Dan Wang, Qin Zhang, Yue Shi, Haiyong Ding, Fei Li

**Affiliations:** ^1^School of Physical Education and Sport Training, Shanghai University of Sport, Shanghai, China; ^2^School of Kinesiology, Shanghai University of Sport, Shanghai, China

**Keywords:** eccentric strength, isometric mid-thigh pull, running performance, neuromuscular characteristics, reactive strength

## Abstract

Neuromuscular characteristics, such as lower-limb joint strength and the ability to rapidly generate force, may play an important role in leg-spring stiffness regulation. This study aimed to investigate the relationship between isokinetic knee and ankle joint peak torque (PT), the force-time characteristics of isometric mid-thigh pull (IMTP), and leg stiffness (*K*_leg_)/vertical stiffness (*K*_vert_) in recreationally trained runners. Thirty-one male runners were recruited and underwent three separate tests. In the first session, the body composition, *K*_leg_, and *K*_vert_ at running speeds of 12 and 14 km⋅h^–1^ were measured. In the second session, isokinetic knee and ankle joint PT at 60°⋅s^–1^ were tested. The force-time characteristics of the IMTP were evaluated in the final session. Pearson’s product-moment correlations, with the Benjamini–Hochberg correction procedure, showed that the knee flexor concentric and eccentric and extensor concentric PT (*r* = 0.473–0.654, *p* < 0.05) were moderate to largely correlated with *K*_leg_ and *K*_vert_ at 12 and 14 km⋅h^–1^. The knee extensor eccentric PT (*r* = 0.440, *p* = 0.050) was moderately correlated with the 14 km⋅h^–1^ *K*_vert_. The ankle plantar flexor concentric and dorsiflexor eccentric PT (*r* = 0.506–0.571, *p* < 0.05) were largely correlated with *K*_leg_ at 12 km⋅h^–1^. The ankle plantar flexor concentric and eccentric and dorsiflexor eccentric PT (*r* = 0.436–0.561, *p* < 0.05) were moderate to largely correlated with *K*_vert_ at 12 and 14 km⋅h^–1^. For IMTP testing, high correlation was only found between the IMPT peak force (PF) and *K*_vert_ at 14 km⋅h^–1^ (*r* = 0.510, *p* = 0.014). Thus, superior leg-spring stiffness in recreational runners may be related to increased knee and ankle joint strength, eccentric muscular capacity, and maximal force production.

## Introduction

The spring-mass model parameters, such as leg stiffness (*K*_leg_) and vertical stiffness (*K*_vert_), are typically used to characterize the mechanical properties of a “leg-spring,” representing the lower limbs during running (Jaén-Carrillo et al., 2021). *K*_leg_ is defined as the ratio between the maximal vertical ground reaction force (vGRF) and the maximum compression in the lower-limb length (Farley and González, 1996). *K*_vert_ describes the global compression of the runner as the ratio of changes in the ground reaction force, to the respective vertical displacement of the center of mass (COM) (Struzik et al., 2021). Greater leg-spring stiffness would facilitate the storage and release of elastic energy, which could reduce the metabolic cost of running (Moore et al., 2019). Previous cross-sectional studies suggested that leg-spring stiffness is largely correlated with running economy (Li et al., 2021) and that endurance-trained athletes express higher leg-spring stiffness than untrained individuals (Hobara et al., 2010). Thus, exploring the principal factors that influence *K*_leg_ and *K*_vert_ is important for optimizing endurance performance.

Neuromuscular strength is an important factor in determining leg-spring stiffness. Improvements in *K*_leg_ and *K*_vert_ after training (e.g., sprint or plyometrics training) are mostly due to neuromuscular adaptation (Ache-Dias et al., 2018; Lum et al., 2019). It has been suggested that muscles, especially at the knee and ankle joints, play an important role in running kinematics and stiffness (Fletcher et al., 2010; Ache-Dias et al., 2018). For example, during the stance braking phase, the knee extensor muscles are the largest contributor to both braking and support of the body mass center (COM) (Hamner et al., 2010). The plantar flexor muscles also produce forces of up to 12 times the body weight during running (Komi, 1990) and are the main force producers during the propulsive phase among all the major lower-limb muscle groups (Monte et al., 2020). Meanwhile, as the running speed increases, the muscle fibers in the ankle plantar flexors [i.e., gastrocnemius medialis (GM)] remain relatively isometric and absorb most of the mechanical power, thereby facilitating greater storage and recovery of tendon elastic strain energy (Lai et al., 2018; Monte et al., 2020). The knee flexor muscles [i.e., vastus lateralis (VL)] are activated to decelerate and support the COM during the stance phase (Liu et al., 2008; Hamner and Delp, 2013) and help the force production of knee flexor muscles at the propulsive phase (Liu et al., 2008; Monte et al., 2020). Therefore, neuromuscular characteristics, such as ankle and knee joint strength, may play an important role in leg-spring stiffness regulation.

However, the relationship between knee and ankle strength and leg-spring stiffness is a topic of debate in scientific and sports communities. As current literature commonly used the ultrasonography technique to measure tendon stiffness or tendinous tissues stiffness, which cannot directly reflect leg-spring stiffness during running. For example, Kubo et al. (2010) found significant correlations between knee extensor strength and tendon stiffness in an untrained population but no significant correlation in the case of well-trained distance runners. Holzer et al. (2018) found that ankle plantar flexor muscle strength was correlated with Achilles tendon stiffness (*r* = 0.50) in older adults, whereas Hirayama et al. (2017) observed that changes in Achilles tendon stiffness were not significantly correlated with changes in plantar flexion strength in healthy men. Therefore, further investigations are needed to examine the relationship between the knee and ankle joint strength and *K*_leg_/*K*_vert_ during practical running.

The runners’ ability to generate force and power within a transitory period during landing could effectively regulate leg-spring stiffness (Lum et al., 2020). The isometric mid-thigh pull (IMTP) is an effective laboratory test that accurately and reliably measures force-time characteristics, such as peak force (PF) generation and rate of force development (RFD), across specific time (Brady et al., 2019). The values obtained from the IMTP test correlate well with powerful athletic abilities, such as sprinting, agility, and jumping performance (Brady et al., 2019). Running involves multiple joints and lower-limb muscle activation (Novacheck, 1998), and IMPT allows the hip, knee, and ankle joints to be at relatively specific angles during running. Thus, it is essential to determine the correlation between the entire lower-limb force generation capacity and leg-spring stiffness. To date, Lum et al. (2020) reported that the IMTP PF and RFD at 0–150 ms were significantly correlated with *K*_leg_ at 12 km⋅h^–1^. Notably, the time required to make contact with the ground and propel the body forward is >200 ms at sub-maximal speed (12 km⋅h^–1^) (Gómez-Molina et al., 2017; García-Pinillos et al., 2019). It is, therefore, important to investigate the force over 200 ms for a comprehensive understanding of the relationship between force-time characteristics and leg-spring stiffness.

Thus, in the present study, we aimed to evaluate the relationship between isokinetic lower-limb joint peak torque (PT) (such as knee and ankle joint concentric and eccentric PT) and IMTP force-time characteristics (such as PF, specific time force at 50–350 ms, specific time RFD at 0–50 to 0–350 ms), and leg-spring stiffness (*K*_leg_ and *K*_vert_) in recreational male runners.

## Materials and Methods

### Participants

Thirty-one recreationally trained male runners (age 20–23 years) from a collegiate running club volunteered to participate in this study. The basic information and physical characteristics of the subjects are presented in Table 1. All participants had previously competed 5–21-km races at the collegiate level with a minimum of 2 years of distance running experience. Before the study, each subject completed the Physical Activity Readiness Questionnaire (PAR-Q). They had to meet the following inclusion criteria: (1) responded “no” to all the PAR-Q questions, (2) trained to run the specified distance (5–21 km) for at least 1 y; (3) ran ≥ 30 km per week for 3 months before the experiment; and (4) no resistance training for 6 months immediately before the beginning of the study. The subjects were fully informed about the procedures and potential risks of the study, and all signed an informed consent document.

**TABLE 1:** Physiological, biomechanical, and neuromuscular strength characteristics of the participants (*n* = 31).

Variables	*Mean*±*SD*	Variables	*Mean*±*SD*
*Physical and physiological characteristics*		*Biomechanical characteristics*	
Age (years)	21 ± 1	Lower limb length (m)	0.94 ± 0.04
Height (cm)	180.6 ± 6.0	Δy at 12 km⋅h^–1^ (cm)	7.38 ± 0.76
Weight (kg)	73.3 ± 9.8	Δy at 14 km⋅h^–1^ (cm)	7.07 ± 0.75
BMI (kg⋅m^–2^)	22.4 ± 1.9	ΔL at 12 km⋅h^–1^ (cm)	15.87 ± 1.27
FFM (kg)	61.8 ± 7.1	ΔL at 14 km⋅h^–1^ (cm)	16.95 ± 1.45
FM (kg)	11.5 ± 4.0	*T*_c_ at 12 km⋅h^–1^ (s)	0.23 ± 0.01
***IMTP force-time characteristics***		*T*_c_ at 14 km⋅h^–1^ (s)	0.22 ± 0.01
PF (N)	1823.74 ± 341.43	vGRF at 12 km⋅h^–1^ (N)	1974.99 ± 253.11
*F*_50_ (N)	338.59 ± 155.14	vGRF at 14 km⋅h^–1^ (N)	2054.03 ± 220.75
*F*_100_ (N)	686.50 ± 205.41	*K*_leg_ at 12 km⋅h^–1^ (kN⋅m^–1^)	12.48 ± 1.52
*F*_150_ (N)	1056.89 ± 244.17	*K*_leg_ at 14 km⋅h^–1^ (kN⋅m^–1^)	12.16 ± 1.28
*F*_200_ (N)	1321.01 ± 267.74	*K*_vert_ at 12 km⋅h^–1^ (kN⋅m^–1^)	26.89 ± 3.38
*F*_250_ (N)	1366.63 ± 226.72	*K*_vert_ at 14 km⋅h^–1^ (kN⋅m^–1^)	29.26 ± 3.63
*F*_300_ (N)	1396.94 ± 222.88	***Isokinetic strength characteristics***	
*F*_350_ (N)	1452.25 ± 233.01	*K*_flex–con_ at 60°⋅s^–1^ (N⋅m)	132.42 ± 19.96
RFD_50_ (N⋅s^–1^)	6430.35 ± 3086.60	*K*_ex–con_ at 60°⋅s^–1^ (N⋅m)	239.10 ± 44.47
RFD_100_ (N⋅s^–1^)	6694.25 ± 2049.00	*K*_flex–ecc_ at 60°⋅s^–1^ (N⋅m)	150.87 ± 27.98
RFD_150_ (N⋅s^–1^)	6932.09 ± 1626.76	*K*_ex–ecc_ at 60°⋅s^–1^ (N⋅m)	274.10 ± 60.86
RFD_200_ (N⋅s^–1^)	6519.68 ± 1339.68	*A*_dors–con_ at 60°⋅s^–1^ (N⋅m)	37.06 ± 6.48
RFD_250_ (N⋅s^–1^)	5398.24 ± 907.68	*A*_plan–con_ at 60°⋅s^–1^ (N⋅m)	128.19 ± 23.73
RFD_300_ (N⋅s^–1^)	4599.54 ± 744.23	*A*_dors–ecc_ at 60°⋅s^–1^ (N⋅m)	59.16 ± 8.37
RFD_350_ (N⋅s^–1^)	4100.50 ± 665.89	*A*_plan–ecc_ at 60°⋅s^–1^ (N⋅m)	223.55 ± 45.25

*BMI, body mass index; FFM, fat-free mass; FM, fat mass; Δy, displacement of the center of mass; ΔL, change in vertical lower limb length; T_c_, ground contact time; vGRF, vertical ground reaction force; K_leg_, leg stiffness; K_vert_, vertical stiffness; K_flex–con_, knee flexor muscles peak torque at in concentric action; K_ex–con_, knee extensor muscles peak torque in concentric action; K_flex–ecc_, knee flexor muscles peak torque in eccentric action; K_ex–ecc_, knee extensor muscles peak torque in eccentric action; A_dors–con_, dorsiflexor muscles peak torque in concentric action; A_plan–con_, plantar flexor muscles peak torque in concentric action; A_dors–ecc_, dorsiflexor muscles peak torque in eccentric action; A_plan–ecc_, plantar flexor muscles peak torque in eccentric action; PF, peak force; F_50_, force at 50 ms; F_100_, force at 100 ms; F_150_, force at 150 ms; F_200_, force at 200 ms; F_250_, force at 250 ms; F_300_, force at 300 ms; F_350_, force at 350 ms; RFD, rate of force development; RFD_0–50_, RFD at 0–50 ms; RFD_0–100_, RFD at 0–100 ms; RFD_0–150_, RFD at 0–150 ms; RFD_0–200_, RFD at 0–200 ms; RFD_0–250_, RFD at 0–250 ms; RFD_0–300_, RFD at 0–300 ms; RFD_0–350_, RFD at 0–350 ms.*

### The Experimental Approach to the Problem

This was a cross-sectional study involving recreational runners to investigate the association between leg-spring stiffness and isokinetic lower-limb joint strength, and isometric time force characteristics. Each subject performed three separate test sessions in the laboratory with a minimum rest interval of 48 h. Before each session, participants were asked to refrain from strenuous exercise for 24 h and acquire more than 8 h of adequate sleep; it was also ensured that they did not experience any muscle soreness or fatigue. The researcher examined the subjects’ commitment to the instructions with the help of the questionnaire when the subjects came to the laboratory for testing. In the first session, the body composition, *K*_leg_, and *K*_vert_ at running speeds of 12 and 14 km⋅h^–1^ were measured. In the second session, PT of concentric and eccentric action at 60°⋅s^–1^ velocity of isokinetic knee flexor and extensor muscles, and that of ankle plantar flexor and dorsiflexor muscles, was conducted. Force-time characteristics (PF, time-specific force at 50–350 ms and RFD within 50–350 ms) of the IMTP were evaluated in the final session.

### Procedures

#### Body Composition Test

Body composition was measured prior to the leg-spring stiffness test. Subjects were required to achieve a rested and hydrated state on the day before the body composition test. On the measurement day, they were required to avoid drink and food intake 2 h before testing. Standing height was measured with a wall-mounted measuring device (Butterfly, Shanghai, China), and the measurement accuracy was within 0.1 cm. Bodyweight, fat mass, and fat-free were measured using a bioimpedance analyzer (X-scan Plus II; Jawon, South Korea). Body mass index (BMI) was calculated by dividing weight in kilograms by height in square meters.

#### Leg-Spring Stiffness Test

**FIGURE 1:**
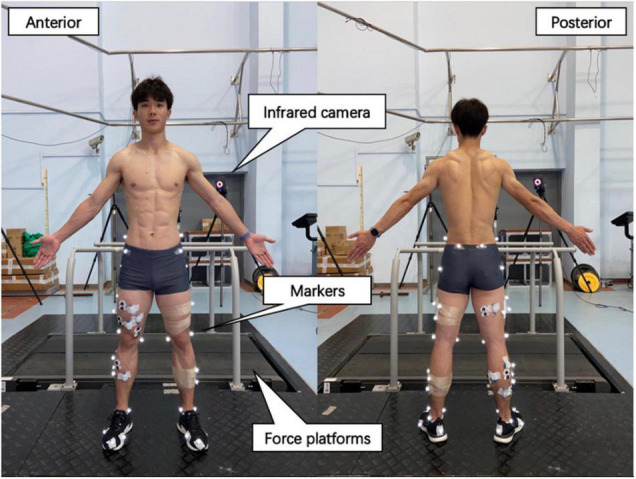
Placement of reflective markers on the leg and experimental site layout. Reflective markers were placed on the following locations to define the hip, thigh, and foot: iliac crest, anterior superior iliac spine, posterior superior iliac spine, femur greater trochanter, femur lateral, and medial epicondyle, fibula apex of the lateral malleolus, tibia apex of the medial malleolus, first and fifth metatarsal heads, posterior surface of the calcaneus, and the surface of the toe. Markers in the T stand were used as tracking thigh and shank.

The participants were required to wear identical running shoes and tight pants before the test to avoid this variable influencing leg-spring stiffness (Kulmala et al., 2018). Reflective markers were placed on the participants’ pelvis and lower limbs to define the pelvis, thigh, shank, and foot segments. The marker locations are shown in Figure 1. The running protocol was performed on a motorized treadmill. The subjects were experienced at running on a treadmill. After 4 min of warm-up at 8 km⋅h^–1^, the subjects performed running at speeds of 12 and 14 km⋅h^–1^ for 4 min, during which time data were recorded for analysis. An 8-camera, 3-dimensional (3D) motion capture system (Vicon T40; Oxford Metrics, Oxford, United Kingdom) was used to record the position of 36 reflective markers (100 Hz) to accurately obtain the vertical displacement of the COM [calculated by the body segmental analysis technique (Gard et al., 2004)] and lower-limb length data [the distance from the greater trochanter of the femur to the ankle during static upright stance (Wurdeman et al., 2017)]. Two 90 cm × 60 cm × 10 cm Kistler 3D force platforms (9287 B, Kistler Corporation) were mounted below the treadmill belt to acquire vGRF data, with a sampling frequency of 1,000 Hz. The vGRF data were synchronized with a 3D motion capture system.

Kinematic data and vGRF were analyzed using the gait analysis software, Visual 3D (v5, C-Motion, Inc., Germantown, MD, United States), using inverse dynamics. The vGRF was filtered with a cut-off frequency of 200 Hz, and the marker trajectories were filtered with a cut-off frequency of 7 Hz using a fourth-order Butterworth low-pass filter (Fu et al., 2017). Based on previous research, recording more than six strides was sufficient to acquire representative data on healthy adults (defined as 95% CIs within 5% error) (Besser et al., 1999). Therefore, we took the data for 10 consecutive strides (beginning at the third minute of each running speed). The average values were calculated for further analysis. A vertical force signal of 50 N was used to identify the beginning and end of contact (Kyröläinen et al., 2001).

Leg stiffness (*K*_leg_) and vertical stiffness (*K*_vert_) were calculated according to Morin’s method (Morin et al., 2005). Pappas et al. (2014) confirmed that the *K*_leg_ and *K*_vert_ measurements obtained during treadmill running using the sine-wave method were highly reliable, exhibiting an intraclass correlation coefficient (ICC) between 0.86 and 0.99. *K*_leg_ (kN⋅m^–1^) is defined as the ratio between the maximal vGRF and the maximum compression in the lower-limb length (Farley and González, 1996) and was calculated as follows:


(1) $$K_{l\,e\,g}=\frac{F_{m\,a\,x}}{△\,L}.$$

(2) $$△\,L=L-\sqrt{L^{2}-{\left(\frac{v\,t_{c}}{2}\right)}^{2}}+△\,y.$$

In this study, *F*_max_ is the maximum vGRF, ΔL is the maximal change in vertical leg length during the stance phase, L is the lower-limb length (the distance from the greater trochanter of the femur to the ankle during static upright stance), Δy is the maximal displacement of the COM during ground contact, v is the running velocity (m⋅s^–1^), and *t*_*c*_ is the ground contact time (s) (a threshold of 50 N vGRF was set when the foot contacted the force plate).

*K*_vert_ (kN⋅m^–1^) is the ratio of the maximum vGRF to the vertical displacement when the COM reaches its lowest point (Farley and González, 1996) and was calculated as follows:


(3) $$K_{v\,e\,r\,t}=\frac{F_{m\,a\,x}}{△\,y}.$$

In this study, *F*_max_ is the maximum vGRF, and Δy is the maximal displacement of the COM during ground contact.

#### Isokinetic Strength Test

**FIGURE 2:**
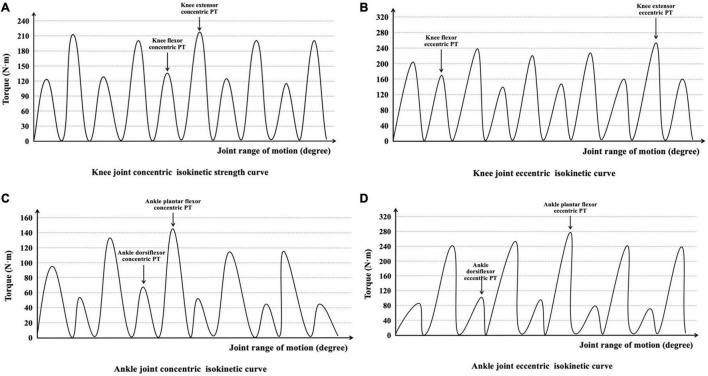
Interpretation of **(A)** knee joint concentric isokinetic strength curve, **(B)** knee joint eccentric isokinetic curve, **(C)** ankle joint concentric isokinetic curve, and **(D)** ankle joint eccentric isokinetic curve during the isokinetic strength test.

The muscles around the knee and ankle joints may play an important role in regulating leg-spring stiffness (Novacheck, 1998; Bohm et al., 2019). PT of the right knee joint flexor/extensor muscles and ankle joint plantar flexor/dorsiflexor muscles was measured using a motor-driven dynamometer (D&R Ferstl GmbH, Hemau, Germany), which showed high ICC (knee: 0.90–0.96; ankle: 0.77–0.98) in previous studies (Gonosova et al., 2018; Andrade et al., 2021). Before each test, the equipment was calibrated according to the instructions of the manufacturer. During the gravity compensation procedure, the shaft of the dynamometer needs to be placed in the basic horizontal position, and the operator should not touch the dynamometer with their hands. The subjects stayed relaxed with no muscle activity during the weighing of the test limbs to eliminate the influence of gravity. All participants performed three warm-up trials using sub-maximum effort to familiarize themselves with the test movements. The subjects then performed five concentric and eccentric isokinetic contractions at angular velocities of 60°⋅s^–1^ for each knee and ankle joint, which could reliably and accurately evaluate PT (Larsson et al., 2006; Silva et al., 2018; Andrade et al., 2021). The PT was calculated as the greatest torque value during isokinetic concentric and eccentric phases and was collected for further analysis (Figure 2). There was a 1-min rest between contraction type and 10-min rest between each joint isokinetic strength test. Additional verbal encouragement was provided during the test.

In the knee joint isokinetic strength test, the subjects sat on the testing table with the knee at 90° flexion. The shaft of the dynamometer was visually aligned with the lateral condyle of the femur and remained constant during the concentric and eccentric contractions (Ehrström et al., 2018). The shoulder and distal femur were then fixed with instruments, and a safety belt and standard stabilization straps were used across the thigh and pelvis to minimize extraneous hip motions. During the test, subjects were instructed to grasp the handles and keep their trunk in contact with the backrest to keep their posture stable. The right knee range of motion was set to begin from full extension to 90° flexion (Sundby and Gorelick, 2014). The subjects were asked to kick and bend the leg as hard and fast as possible throughout a complete range of motion. The knee flexors were measured concentric (*K*_flex–con_), eccentric (*K*_flex–ecc_), knee extensor concentric (*K*_ex–con_), and eccentric (*K*_ex–ecc_) PT at 60°⋅s^–1^.

In the ankle joint isokinetic strength test, subjects laid supine on the dynamometer seat with their hips and knees fully extended, and the right foot was placed on the foot plate. The shaft of the dynamometer was visually aligned with the lateral malleolus of the foot and kept constant during the concentric and eccentric contractions (Svoboda et al., 2019). The right foot was secured by two straps placed across the dorsum of the foot. The thigh, hip, and shoulder were also fixed. The ankle neutral position was set to 0°, and the range of ankle movement started from 15° dorsiflexion to 40° plantar flexion (Tsiokanos et al., 2002). All settings remained the same throughout the experiment. The ankle dorsiflexors concentric (*A*_dors–con_), eccentric (*A*_dors–ecc_), plantar flexor concentric (*A*_plan–con_), and eccentric (*A*_plan–ecc_) PT were measured at 60°⋅s^–1^.

#### Isometric Mid-Thigh Pull Test

The IMTP test was performed using a portable test rack and two force platforms at a sampling frequency of 1,000 Hz (9290AA; Kistler, Winterthur, Switzerland). This test has consistently proven to be highly reliable for ICCs ranging from 0.80 to 0.97 (McGuigan, 2019). Before the test, the mid-thigh position between the hip and knee joints was marked. The subject was instructed to place the bar in the clean second pull position, which was defined by 125–145° of knee flexion and 140–150° of hip flexion (Chavda et al., 2020). All subjects were asked to use an overhand grip secured to the barbell with athletic tape to eliminate the effect of grip strength. Four warm-up trials were performed using 50 and 75% of their maximum effort with a rest period of 60 s (Carroll et al., 2018). Before the test, the subjects were asked to “Push the ground fast and hard with maximum effort” to be at their best. Two maximum-effort IMTP tests were then completed with a 3-min interval between each trial. Participants were encouraged to complete the tests with maximum effort.

Force-time curves were collected at rest for 2 s and at full pull for 5 s during the IMTP test, with the highest force generated reported as the absolute PF. In addition, the time-specific forces at 50–350 ms (*F*_50,_ *F*_100,_ *F*_150,_ *F*_200,_ *F*_250,_ *F*_300,_ and *F*_350_) from the initiation of the pull were collected for every trial. The calculation formula for the RFD is:


(4) R⁢F⁢D=Δ⁢F⁢o⁢r⁢c⁢e/Δ⁢T⁢i⁢m⁢e.

**FIGURE 3:**
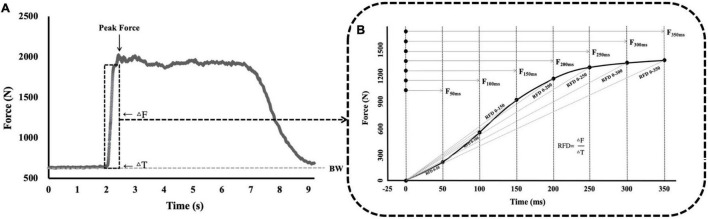
Commonly quantified variables during the analysis of the IMTP force-time curve. **(A)** The IMTP force-time curve at 0–9 s. **(B)** The IMTP force-time curve at 0–350 ms. BW, basic weight; Δ*F*, change in force; Δ*T*, change in time; PF, peak force; *F*_50_, force at 50 ms; *F*_100_, force at 100 ms; *F*_150_, force at 150 ms; *F*_200_, force at 200 ms; *F*_250_, force at 250 ms; *F*_300_, force at 300 ms; *F*_350_, force at 350 ms; RFD, rate of force development; RFD_0–50_, RFD at 0–50 ms; RFD_0–100_, RFD at 0–100 ms; RFD_0–150_, RFD at 0–150 ms; RFD_0–200_, RFD at 0–200 ms; RFD_0–250_, RFD at 0–250 ms; RFD_0–300_, RFD at 0–300 ms; RFD_0–350_, RFD at 0–350 ms; IMTP, isometric mid-thigh pull; RFD, rate of force development.

The RFD was applied to pre-determined time bands: 0–50 to 0–350 ms (RFD_0–50__ms_, RFD_0–100__ms_, RFD_0–150__ms_, RFD_0–200__ms_, RFD_0–250__ms_, RFD_0–300__ms_, and RFD_0–350__ms_) (Figure 3). These time intervals were selected based on the ground contact times at various sub-maximum running speeds (Gómez-Molina et al., 2017; García-Pinillos et al., 2019).

### Statistical Analyses

The Shapiro–Wilk test was used to test data normality. Values are expressed as mean ± SD. The correlations between isokinetic strength (knee and ankle joint concentric and eccentric PT), IMTP force-time characteristics (PF, specific time force at 50–350 ms, specific time RFD at 0–50 to 0–350 ms), and leg-spring stiffness (*K*_leg_ and *K*_vert_ at 12 and 14 km⋅h^–1^) were analyzed using Pearson’s product-moment correlations. Magnitudes of correlation (*r*) were defined as “small” when *r* = 0.1–0.3, “moderate” when *r* = 0.3–0.5, “large” when *r* = 0.5–0.7, “very large” when *r* = 0.7–0.9, and “extremely large” when *r* = 0.9–1.0 (Hopkins et al., 2009). All reported values for *p* were controlled for false-discovery rates using the Benjamini–Hochberg procedure (Benjamini and Hochberg, 1995). Statistical significance was set at *p* < 0.05. All statistical analyses were performed using SPSS (version 22.0; IBM Corp. Armonk, NY, United States) and the statistical programming language R.^1^

## Results

All isokinetic strength, IMTP time force characteristics, and leg-spring stiffness values are listed in Table 1. The correlation coefficients between the neuromuscular indicators and leg-spring stiffness (*K*_leg_ and *K*_vert_ at 12 and 14 km⋅h^–1^) are shown in Table 2.

**TABLE 2:** Correlations between Neuromuscular characteristics and leg-spring stiffness at running speed of 12 and 14 km⋅h^–1^ (*n* = 31).

	*K*_leg_ at 12 km⋅h^–1^		*K*_leg_ at 14 km⋅h^–1^		*K*_vert_ at 12 km⋅h^–1^		*K*_vert_ at 14 km⋅h^–1^	
	Correction coeffient (R)	*Q* value (corrected *p* value)	Correction coeffient (R)	*Q* value (corrected *p* value)	Correction coeffient (R)	*Q* value (corrected *p* value)	Correction coeffient (R)	*Q* value (corrected *p* value)
*K*_flex–con_ at 60°⋅s^–1^	**0.534**	0.018**	**0.618**	<0.001***	**0.525**	0.014**	**0.640**	<0.001***
*K*_ex–con_ at 60°⋅s^–1^	**0.473**	0.040**	**0.494**	0.033**	0.424	0.056	**0.540**	0.014**
*K*_flex–ecc_ at 60°⋅s^–1^	**0.654**	<0.001***	**0.610**	<0.001***	**0.552**	0.012**	**0.651**	<0.001***
*K*_ex–ecc_ at 60°⋅s^–1^	0.406	0.106	0.449	0.056	0.345	0.131	**0.440**	0.050*
*A*_dors–con_ at 60°⋅s^–1^	0.192	0.445	0.017	0.928	0.406	0.074	0.370	0.108
*A*_plan–con_ at 60°⋅s^–1^	**0.571**	0.012**	0.390	0.115	**0.561**	0.012**	**0.517**	0.014**
*A*_dors–ecc_ at 60°⋅s^–1^	**0.506**	0.031**	0.358	0.147	**0.521**	0.014**	**0.511**	0.014**
*A*_plan–ecc_ at 60°⋅s^–1^	0.313	0.241	0.259	0.297	**0.436**	0.050*	**0.438**	0.050*
PF	0.292	0.241	0.397	0.113	0.383	0.098	**0.510**	0.014**
*F*_50_	0.278	0.260	0.141	0.571	0.260	0.258	0.187	0.370
*F*_100_	0.144	0.571	0.066	0.749	0.229	0.310	0.207	0.329
*F*_150_	0.121	0.592	0.086	0.700	0.177	0.375	0.207	0.329
*F*_200_	0.295	0.241	0.256	0.297	0.292	0.213	0.322	0.169
*F*_250_	0.305	0.241	0.365	0.147	0.274	0.239	0.365	0.110
*F*_300_	0.119	0.592	0.226	0.347	0.119	0.525	0.221	0.325
*F*_350_	0.139	0.571	0.243	0.314	0.132	0.500	0.234	0,310
RFD_50_	0.279	0.260	0.138	0.571	0.262	0.258	0.183	0.374
RFD_100_	0.144	0.571	0.064	0.749	0.229	0.310	0.204	0.329
RFD_150_	0.120	0.592	0.084	0.700	0.177	0.375	0.204	0.329
RFD_200_	0.294	0.241	0.254	0.297	0.292	0.213	0.319	0.169
RFD_250_	0.305	0.241	0.363	0.147	0.274	0.239	0.361	0.111
RFD_300_	0.118	0.592	0.224	0.347	0.119	0.525	0.217	0.326
RFD_350_	0.138	0.571	0.241	0.314	0.132	0.500	0.231	0.310

*Significant correlation (*P < 0.05, **P < 0.01, ***P < 0.001). Significant effects are reported in bold font.

*K_flex–con_, knee flexor muscles peak torque at in concentric action; K_ex–con_, knee extensor muscles peak torque in concentric action; K_flex–ecc_, knee flexor muscles peak torque in eccentric action; K_ex–ecc_, knee extensor muscles peak torque in eccentric action; A_dors–con_, dorsiflexor muscles peak torque in concentric action; A_plan–con_, plantar flexor muscles peak torque in concentric action; A_dors–ecc_, dorsiflexor muscles peak torque in eccentric action; A_plan–ecc_, plantar flexor muscles peak torque in eccentric action.*

*PF, peak force; F_50_, force at 50 ms; F_100_, force at 100 ms; F_150_, force at 150 ms; F_200_, force at 200 ms; F_250_, force at 250 ms; F_300_, force at 300 ms; F_350_, force at 350 ms; RFD, rate of force development; RFD_0–50_, RFD at 0–50 ms; RFD_0–100_, RFD at 0–100 ms; RFD_0–150_, RFD at 0–150 ms; RFD_0–200_, RFD at 0–200 ms; RFD_0–250_, RFD at 0–250 ms; RFD_0–300_, RFD at 0–300 ms; RFD_0–350_, RFD at 0–350 ms.*

**FIGURE 4:**
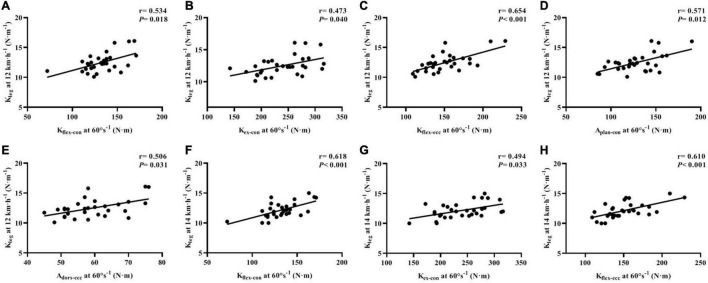
Correlations of isokinetic lower-limb joint strength and IMTP force-time characteristics with leg stiffness at running speeds of 12 and 14 km⋅h^–1^ **(A–H)**. *K*_flex–con_, knee flexor muscles peak torque in concentric action; *K*_ex–con_, knee extensor muscles peak torque in concentric action; *K*_flex–ecc_, knee flexor muscles peak torque in eccentric action; *K*_ex–ecc_, knee extensor muscles peak torque in eccentric action; *A*_plan–con_, plantar flexor muscles peak torque in concentric action; *A*_dors–ecc_, dorsiflexor muscles peak torque in eccentric action; IMTP, isometric mid-thigh pull.

For correlations between the isokinetic strength testing and leg-spring stiffness test, we found that the PT of *K*_flex–con_ (*r* = 0.534, *p* = 0.018; *r* = 0.618, *p* < 0.001, respectively), *K*_flex–ecc_ (*r* = 0.654, *p* < 0.001; *r* = 0.610, *p* < 0.001, respectively), and *K*_ex–con_ (*r* = 0.473, *p* = 0.040; *r* = 0.494, *p* = 0.033, respectively) was moderately to largely correlated with *K*_leg_ at 12 and 14 km⋅h^–1^. The PT of *A*_plan–con_ (*r* = 0.571, *p* = 0.012) and *A*_dors–ecc_ (*r* = 0.506, *p* = 0.031) was largely correlated with 12 km⋅h^–1^ *K*_leg_ after Benjamini–Hochberg adjustment (Figure 4).

**FIGURE 5:**
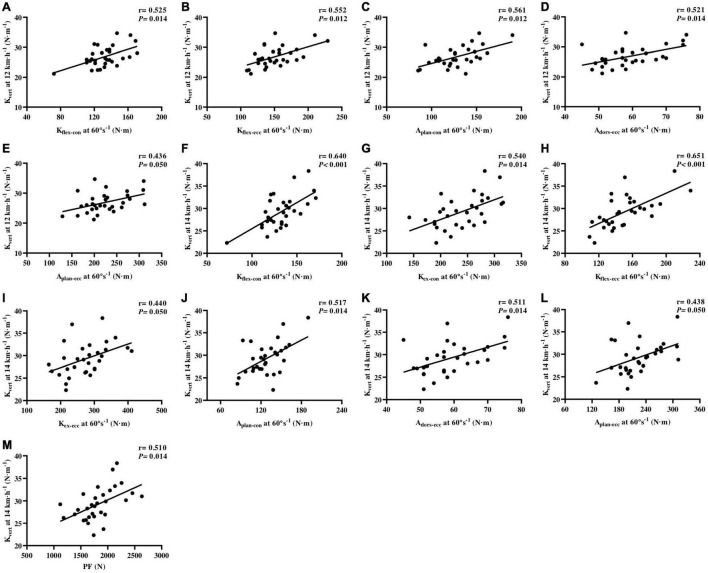
Correlations of isokinetic lower-limb joint strength and IMTP force-time characteristics vertical stiffness at running speeds of 12 and 14 km⋅h^–1^ **(A–M)**. *K*_flex–con_, knee flexor muscles peak torque in concentric action; *K*_ex–con_, knee extensor muscles peak torque in concentric action; *K*_flex–ecc_, knee flexor muscles peak torque in eccentric action; *K*_ex–ecc_, knee extensor muscles peak torque in eccentric action; *A*_plan–con_, plantar flexor muscles peak torque in concentric action; *A*_dors–ecc_, dorsiflexor muscles peak torque in eccentric action; *A*_plan–ecc_, plantar flexor muscles peak torque in eccentric action; PF, peak force; IMTP, isometric mid-thigh pull.

The PT of *K*_flex–con_ (*r* = 0.525, *p* = 0.014; *r* = 0.640, *p* < 0.001, respectively), *K*_flex–ecc_ (*r* = 0.552, *p* = 0.012; *r* = 0.651, *p* < 0.001, respectively), *A*_plan–con_ (*r* = 0.561, *p* = 0.012; *r* = 0.517, *p* = 0.014, respectively), *A*_plan–ecc_ (*r* = 0.436, *p* = 0.050; *r* = 0.438, *p* = 0.050, respectively), and *A*_dors–ecc_ (*r* = 0.521, *p* = 0.014; *r* = 0.511, *p* = 0.014, respectively) was moderately to largely correlated with *K*_vert_ at 12 and 14 km⋅h^–1^. The PT of *K*_ex–con_ (*r* = 0.540, *p* = 0.014) and *K*_ex–ecc_ (*r* = 0.440, *p* = 0.050) was moderately to largely correlated with the 14 km⋅h^–1^ *K*_vert_ (Figure 5).

With regard to IMTP testing, we only found largely relationships between the IMPT PF (*r* = 0.510, *p* = 0.014) and *K*_vert_ at 14 km⋅h^–1^, after Benjamini–Hochberg correction (Figure 5). The specific time force at 50–350 ms and RFD at 0–50 to 0–350 ms were not correlated with *K*_leg_ and *K*_vert_ at 12 and 14 km⋅h^–1^, after false-discovery rate corrections.

## Discussion

**FIGURE 6:**
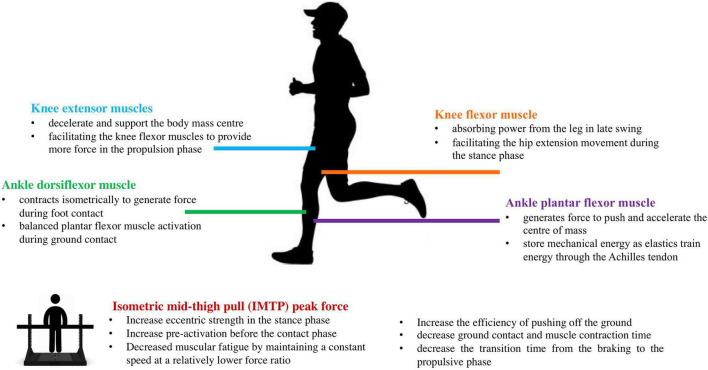
Infographic of the potential mechanisms of the neuromuscular characteristics regulating leg-spring stiffness.

Leg-spring stiffness (*K*_leg_ and *K*_vert_) is an important parameter regulating running mechanics because it reflects the amount of energy storage and recoil for a given compression. However, scientific literature on the relationship of neuromuscular strength with leg-spring stiffness remains unclear. This study aimed to investigate the relationship between isokinetic lower-limb joint strength, isometric force-time characteristics, and leg-spring stiffness in recreationally trained male runners. Our primary finding was that the maximum torque of knee and ankle production was strongly correlated with *K*_leg_ and *K*_vert_. Furthermore, only the IMTP PF was correlated with *K*_vert_ at 14 km⋅h^–1^. No correlations were found between leg-spring stiffness and specific time force at 50–350 ms or RFD at 0–50 to 0–350 ms. The potential mechanisms of how these neuromuscular characteristics regulate leg-spring stiffness are shown in Figure 6.

*K*_leg_ and *K*_vert_ indicated that runners exhibited the capacity to quickly absorb and return mechanical energy during running. In this study, the *K*_leg_ values (12.16–12.48 kN⋅m^–1^) and *K*_vert_ values (26.89–29.26 kN⋅m^–1^) reported are similar to those of previous investigations at similar running speeds (Morin et al., 2005; García-Pinillos et al., 2020), which used the same methods to calculate leg-spring stiffness in runners. Meanwhile, we found that *K*_leg_ and *K*_vert_ had a moderate to large correlation with isokinetic knee strength, indicating that the knee flexor and extensor muscles play important roles in regulating leg-spring stiffness. This finding corroborates the study of Hayes and Caplan (2014) who reported significant associations between knee flexor PT and *K*_leg_ in middle-distance runners. Meanwhile, using the ultrasonography technique, Kubo et al. (2010) reported that patellar tendon stiffness was significantly correlated with knee extensor PT in an untrained population. During the early stance phase, the knee extensor muscles (i.e., VL) are significantly activated to decelerate and support the COM (Bohm et al., 2018). The knee extensor muscle fascicles contract quasi-isometrically, allowing the knee flexor muscles (i.e., hamstrings) to provide more force in the propulsion phase (Monte et al., 2020). The primary knee flexor muscle (i.e., hamstring) performs biarticular muscle functions, absorbing power from the leg in late swing, and facilitating the hip extension movement during the stance phase (Sasaki and Neptune, 2006). As running speed increases, the contribution of VL to force production decreases as a function of speed, allowing the hamstrings to provide more force (Monte et al., 2020). On the other hand, pre-activation before ground contact and a more forceful eccentric action have proven to be critical in regulating leg-spring stiffness (Bonacci et al., 2009; Douglas et al., 2020). Although we did not test the muscle pre-activation profile during initial foot contact, previous research indicated that stronger athletes elicited higher pre-activation of the leg extensor muscles compared to weaker counterparts (Kyröläinen and Komi, 1995). Greater muscle pre-activation appears to resist high impact loads during landing (Kyröläinen and Komi, 1995), and a stronger eccentric force within the braking phase may help to prevent excessive tendon lengthening under high stretch loads and reinforce force output during the propulsive phase *via* the residual force (Fukutani et al., 2017). Therefore, we may conclude that the greater knee flexor and extensor muscle groups work together to regulate the *K*_leg_ and *K*_vert_ by providing stability during the stance phase and enhancing the force generation during the propulsion phase.

For the ankle joint, we found that plantar concentric and eccentric PT and dorsiflexion eccentric PT were significantly correlated with 12 km⋅h^–1^ *K*_leg_ and *K*_vert_ at 12 and 14 km⋅h^–1^. Despite the scarce evidence for the associations between ankle joint strength and leg-spring stiffness, Arampatzis et al. (2006) reported that more economical runners have a higher plantar flexor strength and greater stiffness of the triceps surae. Holzer et al. (2018) found a significant correlation between ankle plantar flexor muscle strength and Achilles tendon stiffness in older adults. In the study of Stenroth et al. (2016), the authors indicated that compared with the young untrained adults (18–30 years old), there was no difference in Achilles tendon stiffness of the older athletes (70–80 years old) competing in endurance or sprint running events, which may attribute to the high-intensity training loading on plantar flexor muscles. In humans, the plantar flexor muscle group is the major contributor to propulsion and generates most of the energy required to push and accelerate the COM (Hamner and Delp, 2013). Meanwhile, the plantar flexion muscles have continuously shortened during the stance phase to produce mechanical work and increase the stretch and recoil of the tendon, thereby facilitating greater storage and recovery of tendon elastic strain energy (Bohm et al., 2019; Monte et al., 2020).

In addition, ankle dorsiflexion eccentric PT was significantly associated with *K*_leg_ and *K*_vert_ scores. One human walking study from Maharaj et al. (2019) pointed out that similar to plantar flexors, the dorsiflexors muscle fascicles [i.e., tibialis anterior (TA)] activated and contracted isometrically to generate force, and the TA tendinous tissue actively lengthened to absorb the energy during foot contact and return it to the body later in the stride. Although the energy that is potentially stored in the TA tendinous tissue is likely to come from that generated by the plantar flexors and recoil of the Achilles tendon (Maharaj et al., 2019). The complex interaction of synergistic antagonist muscles suggests that co-activation of muscles may be useful for tuning the stiffness of a joint, rather than just providing joint stability. Tam et al. (2018) supported this idea by reporting that more economical runners exhibit balanced or equal lateral gastrocnemius and TA muscle activation during ground contact. Overall, our study provides evidence that the knee flexor and extensor concentric and eccentric PT, ankle plantar concentric and eccentric PT, and dorsiflexion eccentric PT are significantly correlated with *K*_leg_ and *K*_vert_ in recreational male runners.

The IMTP is performed to determine the force-time characteristics of various sports and athletes (McGuigan, 2019). Lum et al. (2020) recently reported that the IMTP PF and RFD at 0–150 ms were significantly correlated with *K*_leg_ at 12 km⋅h^–1^, concluding that runners with the capacity to generate force more rapidly exhibit greater leg-spring stiffness. However, the authors did not measure the force over 200 ms in this study. Our data (215–234 ms), and those of previous studies (Gómez-Molina et al., 2017; García-Pinillos et al., 2019), show that the contact time at a running speed of 12–14 km⋅h^–1^ is greater than 200 ms. Therefore, it is necessary to measure the force over 200 ms for recreational runners. In the present study, we only found that the IMTP PF showed a large relationship with 14 km⋅h^–1^ *K*_vert_, supporting the findings of Lum et al. (2020). A larger PF represents a better neuromuscular function that regulates leg-spring stiffness. First, previous research reported a large association (*r* = 0.896) between IMTP PF and eccentric strength (Spiteri et al., 2014). The superior eccentric strength within the stance phase may increase subsequent quasi-isometric and concentric force production during the propulsive phase *via* residual force enhancement and stretch-shortening cycle utilization (Fukutani et al., 2017; Douglas et al., 2020). Beattie et al. (2017) reported a moderate association between maximum-strength variables (IMPT PF) and reactive strength capacity. Second, muscle fatigue is the major mechanism affecting leg-spring stiffness (Dutto and Smith, 2002). Dutto and Smith (2002) found that *K*_leg_ in runners was significantly decreased during a treadmill run-to-exhaustion test. The generation of a greater force by lower-limb muscle contraction allows runners to maintain a constant speed or perform each running action at a relatively lower force ratio (Aagaard and Andersen, 2010). Therefore, it decreased muscular fatigue at a given speed, allowing runners to sustain a higher leg-spring stiffness and reduce metabolic demand. However, we did not find any correlations between the IMPT PF and *K*_vert_ or *K*_leg_ at 12 km⋅h^–1^. This was possible because participants were unlikely to maximally activate their muscles during the lower running speed trials (12 km⋅h^–1^). At a higher speed (14 km⋅h^–1^), the muscle activity during the stance phase may reinforce the driving force in the forward direction (Kyröläinen et al., 2001). Therefore, higher running speeds may correlate with leg-spring stiffness and PF production.

Notably, we did not find any correlations between leg-spring stiffness and specific time force at 50–350 ms or RFD at 0–50 to 0–350 ms. This is inconsistent with the results of Lum et al. (2020); they reported that IMTP RFD at 0–150 ms was significantly correlated with *K*_leg_. One possible explanation is that our recreational cohort rarely performed resistance training to reinforce their explosive strength. As the reliability of data in RFD periods (especially for 50–150 ms) was relatively low (Merrigan et al., 2020), resistance training experience may have led to an unstable IMPT value. In addition, previous studies indicated that with the increase in the running speed, both the average vGRF (Hamill et al., 1983) and vertical average loading rate (the slope of the first vGRF from 20 to 80% of the stance time) (Yu et al., 2021) will increase. Additionally, the running speeds in the present study were relatively low (12 and 14 km⋅h^–1^) and did not require explosive contraction, which may have resulted in a limited correlation with leg-spring stiffness. By contrast, the PF of IMPT was largely correlated with vertical stiffness, as *K*_vert_ was calculated using *F*_max_. Although no statistical correlation was found, the ability to quickly generate a higher force is essential for runners. Theoretically, it enables runners to rapidly push off the ground, thereby decreasing ground contact and resulting in a quicker transition from the braking to the propulsive phase of the running gait (Lum et al., 2020), as 90–96% of the variance in *K*_leg_ stiffness can be distinguished by changing the contact time during running (Morin et al., 2007). Runners with shorter contact times may potentially reduce vertical oscillation (Lima and Blagrove, 2020) and regulate leg-spring stiffness through neuromuscular factors.

This study has certain limitations. First, current findings indicate that the strength mentioned above is an acceptable predictor of leg-spring stiffness in recreational runners. However, further studies, such as those involving electromyography during running, are needed to determine the direct neuromuscular indicators (e.g., pre-activation before ground contact). In addition, a large correlation does not imply a cause-effect relationship. Therefore, studies with a longitudinal study design should be performed in the future to confirm the effects of isokinetic joint strength, isometric force-time characteristics, and their manipulation through intervention on leg-spring stiffness.

## Conclusion

In summary, we found that the knee flexor and extensor concentric/eccentric PT, ankle plantar flexor concentric and eccentric PT, and dorsiflexor eccentric PT at 60°⋅s^–1^ were significantly correlated with *K*_leg_ and *K*_vert_. Furthermore, the IMTP PF was largely correlated with *K*_vert_ at 14 km⋅h^–1^. Therefore, leg-spring stiffness is associated with specific neuromuscular characteristics in recreational runners. Runners should focus on improving their knee and ankle joint strength, eccentric muscular capacity, and maximal force production. Although we did not find a correlation between specific time interval force or RFD and leg-spring stiffness, a previous study indicated that quicker force production facilitates leg-spring stiffness regulation (Lum et al., 2020; Li et al., 2021). Therefore, exercises such as resistance, power, and plyometric training may be used to increase leg-spring stiffness and running performance.

## Data Availability Statement

The raw data supporting the conclusions of this article will be made available by the authors, without undue reservation.

## Ethics Statement

The studies involving human participants were reviewed and approved by Ethics Committee of Shanghai University of Sport, China (ID number: 2017047). The participants provided written informed consent to participate in this study. Written informed consent was obtained from the participants for the publication of any potentially identifiable images or data included in this manuscript.

## Author Contributions

SC and QZ conceived and designed the experiments, performed the experiments, analyzed the data, contributed reagents, materials, and analysis tools, prepared figures and/or tables, authored or reviewed drafts of the manuscript, and approved the final draft. DW conceived and designed the experiments, and authored or reviewed drafts of the manuscript. YS performed the experiments, and prepared figures and/or tables. HD guided the writing of the manuscript, critically revised the manuscript, and approved the final draft. FL conceived and designed the experiments, and approved the final draft. All authors contributed to the article and approved the submitted version.

## Conflict of Interest

The authors declare that the research was conducted in the absence of any commercial or financial relationships that could be construed as a potential conflict of interest.

## Publisher’s Note

All claims expressed in this article are solely those of the authors and do not necessarily represent those of their affiliated organizations, or those of the publisher, the editors and the reviewers. Any product that may be evaluated in this article, or claim that may be made by its manufacturer, is not guaranteed or endorsed by the publisher.

## Footnotes

1
www.r-project.org
